# Tear film osmolarity variation between weeks in healthy and dry eye disease subjects

**DOI:** 10.5935/0004-2749.2022-0043

**Published:** 2023-03-08

**Authors:** Hugo Pena-Verdeal, Jacobo Garcia-Queiruga, Belen Sabucedo-Villamarin, María J. Giraldez, Eva Yebra-Pimentel

**Affiliations:** 1 Departamento de Física Aplicada, Área de Optometría, Universidade de Santiago de Compostela, Santiago de Compostela (Galicia), Spain

**Keywords:** Osmolar concentration, Tears, Dry eye syndromes, Diagnostic techniques, ophthalmological, Concentração osmolar, Lágrimas, Síndromes do olho seco, Técnicas de diagnóstico oftalmológico

## Abstract

**Purpose:**

The possible variability in diagnostic test results is a statistical feature of dry eye disease patients. The clinician should consider tear film variations over time since the timing of tear film measurements is important for proper diagnosis. The purpose of the present study was to analyze the inter-week variation of osmolarity measurement in healthy and dry eye disease participants.

**Methods:**

Based on the Dry Eye Workshop II (DEWS-II) diagnostic methodology report criteria, a battery of tests (Ocular Surface Disease Index [OSDI] questionnaire, breakup time, and corneal staining) was administered to rule out the presence of dry eye disease. A total of 40 qualified volunteers were recruited into two groups: with only 20 healthy and 20 dry eye disease participants. The inter-week variation of osmolarity in the two groups was measured using a TearLab osmometer in two sessions one-week apart. The differences between the results were calculated.

**Results:**

There were no significant differences in osmolarity between the two sessions for either the healthy (paired t-test; p=0.085) or dry eye disease (paired t-test; p=0.093) participants. Moreover, there was no significant correlation between the means and differences in either session on healthy (Pearson correlation: r=0.020; p=0.935) or dry eye disease (Pearson correlation: r=-0.022; p=0.928) participants. In session 1, there was a significant difference in osmolarity values between groups (unpaired t-test; p=0.001), but no difference was found in session 2 (unpaired t-test; p=0.292).

**Conclusions:**

The present study discovered no inter-week variation in the tear film osmolarity of healthy and dry eye disease participants classified based on the DEWS-II criteria.

## Abstract (pt)

**Objetivo:**

A possível variabilidade nos resultados de testes diagnósticos é uma característica estatística dos pacientes com síndrome do olho seco. O médico deve considerar as variações do filme lacrimal ao longo do tempo, pois o momento em que o filme lacrimal é medido pode ser crítico para o diagnóstico adequado. O objetivo deste estudo foi analisar a variação semanal da osmolaridade do filme lacrimal em participantes saudáveis e em outros com síndrome do olho seco.

**Métodos:**

Com base nos critérios da metodologia de diagnóstico do relatório da *Dry Eye Workshop II* (DEWSII), foi aplicada uma bateria de testes (questionário do índice de doença da superfície ocular [OSDI], tempo de ruptura do filme lacrimal e coloração da córnea) para descartar a presença de síndrome do olho seco. Um total de 40 voluntários qualificados foi recrutado e distribuído em dois grupos de 20 participantes saudáveis e 20 participantes com síndrome do olho seco. A variação da osmolaridade entre semanas foi medida com um osmômetro TearLab em duas sessões com uma semana de intervalo nos dois grupos. As diferenças entre os resultados foram então calculadas.

**Resultados:**

Não foram encontradas diferenças significativas na osmolaridade entre as medidas obtidas nas duas sessões, nem no grupo de participantes saudáveis (teste de *t* pareado; p=0,085), nem no de participantes com síndrome do olho seco (teste de *t* pareado; p=0,093). Não foi detectada nenhuma correlação significativa entre as médias e diferenças entre as duas sessões entre participantes saudáveis (correlação de Pearson: r=0,020, p=0,935) e aqueles com síndrome do olho seco (correlação Pearson: r=-0,022, p=0,928). Foi encontrada uma diferença significativa nos valores de osmolaridade entre os dois grupos na primeira sessão (teste de *t* não pareado; p=0,001), mas nenhuma diferença foi encontrada na segunda sessão (teste de *t* não pareado; p=0,292).

**Conclusões:**

O presente estudo não encontrou variação entre semanas consecutivas na osmolaridade do filme lacrimal em participantes saudáveis e com síndrome do olho seco, classificados com base nos critérios do DEWSII.

## INTRODUCTION

Dry eye disease (DED) has been redefined by the Dry Eye Workshop II (DEWS-II) as a multifactorial disease of the ocular surface characterized by a loss of homeostasis of the tear film and accompanied by ocular symptoms, in which tear film instability and hyperosmolarity, ocular surface inflammation and damage, and neurosensory abnormalities play etiological roles^(1)^. Regardless of the underlying etiology, the DEWS-II report reaffirmed that an increase in tear osmolarity is a key mechanism in DED^(2)^. Since elevated tear film osmolarity is thought to be a core mechanism of ocular surface damage and the inherent DED symptomatology, it has been proposed as a gold standard in the diagnosis of dry eye^(3-5)^. Measuring osmolarity allows you to capture the status of the tear film in a single parameter, providing a powerful tool that has even been described as the gold standard for DED diagnosis^(2,3)^.

In dry eye diagnosis, it is important to note that tests are affected by temporal variations, which can have a negative impact on cross-section studies^(6)^. Indeed, because of the heteroscedasticity, variability in test results is a statistical characteristic of DED patients and has been proposed as a clinical indicator of the normal tear film homeostasis loss^(6)^. Clinicians should consider the possibility of variations in tear film parameters over time because the time when measurements are performed can be critical for a proper diagnosis and management. Previous studies examined the variation of osmolarity results for one day or consecutive days, and the results were highly variable^(7-12)^. Therefore, the present study aimed to analyze the inter-week variation of osmolarity measurement in healthy and DED participants classified using DEWS-II criteria.

## METHODS

### Sample characteristics and inclusion criteria

The present study was designed as a continuous dependent response variable based on paired measurements of participants. PS Power and Sample Size Calculations Version 3.1.2 (Copyright © by William D. Dupont and Walton D. Plummer) was used to calculate sample size. According to previous literature^(2)^, the osmolarity mean Standard Deviation (SD) of repeated measures is normally distributed with a mean value of 4.8 mOsm/L, and a difference in the mean response of matched pairs is 5 mOsm/L; to reject the null hypothesis that this response difference is 0 with a probability (power) of 0.80 (the Type I error probability associated with this test was 0.05), a minimum of 12 participants were required to be examined twice. To accomplish a more reliable study, a larger population of 40 qualified participants was recruited and divided into two study groups of 20 participants each. If a subject had a history of a conjunctival, scleral, or corneal disease, active ocular disease or ocular allergy, prior eye surgery (including refractive surgery or eyelid tattooing), glaucoma, diabetes mellitus, thyroid disorder, was pregnant or breastfeeding, wore contact lenses, or had systemic inflammatory/autoimmune disease, they were excluded. At the time of the study, no participant was taking any topical and systemic medications or using artificial tears. After revising the inclusion and exclusion criteria, participants were recruited from patients attending the Optometry Clinic of the center, and participants gave their informed consent. The study protocol followed the principles the Helsinki Decla-ration and was approved by the institution.

Based on DEWS-II criteria^(2)^, a battery of dry eye tests comprised of the Ocular Surface Disease Index (OSDI) questionnaire, breakup time (BUT), and corneal staining was administered to volunteer participants to rule or not rule out DED presence prior to inclusion in this study. Participants were classified as DED (if all of three diagnosis criteria were met, an OSDI score higher than 13, a BUT lower than 10 seconds, and a corneal staining grade higher than 1 on the Oxford Grading) or healthy (if all three diagnosis criteria were met)^(2)^.

### Osmolarity measurement

Tear film osmolarity was determined using the TearLab Osmometer (TearLab, USA), a tear osmometer that requires a 0.05 µl sample taken directly through capillary action by a probe^(7,12-14)^. With the subject seated with the chin tilted upward and eyes directed toward the ceiling until a beep indicated that a tear sample had been collected, the instrument probe (housing the disposable microchip) was placed on the lower tear meniscus. The device converts the electrical impedance of the sample into osmolarity (mOsm/L) in less than 10 seconds, which is displayed on the device screen. It had a measurement range of 275-400 mOsm/L. Quality control electronic check cards provided by the manufacturer were used on a daily basis to verify the correct status of the system according to the given specifications (if the reading was 334 ± 3 mOsm/L, the pen was working properly). In all procedures, the same test card lot number was used.

To minimize possible diurnal variations, osmolarity was measured in two sessions in each subject twice, one--week apart, at the same hour^(7,12,13)^. To avoid overstating the precision of statistical estimates or any possible variation in osmolarity between eyes, all procedures were carried out in one participant’s eye (right eye)^(12,15,16)^. To avoid inter-observer variability in the collecting process, all osmolarity measurements were performed by the same investigator (left-handed)^(17)^. To avoid any possible diurnal variation, all measurements were performed on all participants at the same time of day (between 15:30 and 18:30)^(18)^. During all measurements, the instrument and test cards used for both study visits were kept in the same humidityand temperature-controlled room (temperature 20-23°C, humidity 50-60%)^(19)^.

### Statistical analysis

The data was analyzed using IBM SPSS Statistics v.25 software (SPSS Inc., Chicago, IL). In all tests performed, the significance level was set at a p≤0.05. Prior to any analysis, the normal distribution of the data was tested using the Shapiro-Wilk test^(20)^. The Shapiro-Wilk test revealed that the obtained data had a normal distribution (Shapiro-Wilk: session 1 both groups p≥0.222; session 2 both groups p≥0.423).

For unpaired samples, differences in gender and age distribution were assessed using a Pearson χ^^2^^ test and a paired t-test, respectively^(20)^.

For the intersession variance study, Bland and Altman procedures were used. This method describes the correlation or similarity between two variables by using averages rather than differences^(20,21)^. Thus, the differences between the sets of measurements obtained in the two sessions were assessed. For related samples, differences were assessed using a paired t-test, and 95% limits of repeatability were calculated (Mean Difference ± 1.96xSD differences)^(20)^; limits of agreement (LoA) were also calculated (Mean difference ± 1.96 x SD), as well as the exact 95% Confidence Intervals (95% CI) for Upper and Lower LoA considered as a pair (Mean difference ± c_t0.025_ x SD; Mean difference ± c_t0.975_ x SD)^(20,22)^. To determine whether the differences between sessions were due to osmolarity values, the correlation between means and differences was calculated by the Pearson correlation test^(20)^. Correlation between variables was classified as weak (0.21-0.40), moderate (0.41-0.60), substantial (0.61-0.80), and strong (0.81-1.0).

For the intra-session analysis, differences between the sets of measurements obtained in each session between groups were assessed using a paired t-test for unpaired samples^(20)^.

## RESULTS

Table 1 summarizes the demographics and descriptive statistics of the battery of dry eye tests administered to volunteer participants to rule or not rule out DED presence prior to inclusion in the study. There was no statistical difference between groups in terms of gender distribution (Pearson χ^^2^^ test; p=0.677, Table 1), but there was a statistical difference in terms of age distribution (unpaired t-test; p<0.001, Table 1).

**Table 1:** Demographics and descriptive statistics of battery of dry eye tests administered to volunteer participants to rule or not rule out DED presence before being included on the study. Age values reported in years. OSDI is a non-dimensional variable. BUT values reported in seconds. Corneal staining recoded according to Oxford Scale Grade

	Sex (women/men)	Age (Mean ± SD)	OSDI (Mean ± SD)	BUT (Mean ± SD)	Corneal staining (Median (IQR))
**Healthy**	16 / 4	19.1 ± 1.33	6.38 ± 2.98	16.1 ± 3.77	2 (0)
**DED**	17 / 3	28.2 ± 8.94	23.9 ± 10.93	4.5 ± 1.92	2 (0)

n = 20 subjects per group; DED= Dry eye disease; OSDI= Ocular Surface Disease Index; BUT= Breakup Time; SD= Standard Deviation; IQR= Interquartile Range.

**Figure 1:**
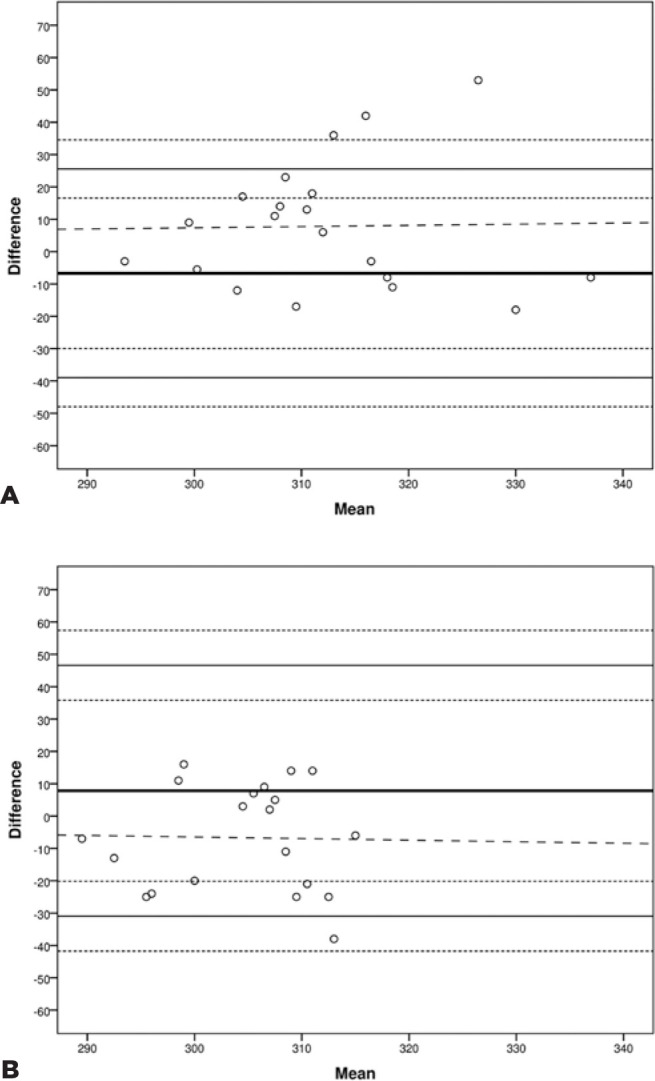
Mean versus differences (Bland-Altman plot) between the values obtained in the two sessions in n = 20 participants. The thick solid horizontal line indicates the mean difference while the thin solid horizontal lines the 95% LoA (Mean difference ± 1.96xSD). The dashed horizontal lines indicate the 95% Confidence Interval of the LoA. A) Healthy group: Session 1 vs. Session 2; B) DED group: Session 1 vs. Session 2. 95% LoA = 95% Limits of Agreement. 95% CI = 95% Confidence Interval.

Table 2 summarizes the mean ± SD for the osmolarity measurements obtained during each session and group. There were no significant differences in measurements between the two sessions for either the healthy (paired t-test; p=0.085) or DED participants (paired t-test; p=0.093) (Table 2). Figure 1 shows Bland and Altman plots of means against differences in data obtained from the two sessions for both groups. There was no significant correlation between the means and differences between both sessions on either healthy (Pearson correlation test: r=0.020, p=0.935) or DED participants (Pearson correlation test: r=-0.022, p=0.928), confirming that the differences between sessions were not dependent on the osmolarity values. However, as shown in Figure 1 and Table 2, the 95% LoA and 95% CI are large in both groups.

**Table 2:** Descriptive statistics, differences and LoA (95%CI) of osmolarity results between groups and measurements recorded in sessions 1 and 2

	Session 1 (Mean ± SD)	Session 2 (Mean ± SD)	Mean difference ± SD	p	Lower LoA (95%CI)	Upper LoA (95%CI)
**Healthy**	301.20 ± 10.89	307.90 ± 11.12	-6.70 ± 16.46	0.085	-38.96 (-29.97 to -47-96)	25.56 (16.57 to 34.56)
**DED**	316.13 ± 14.56	308.30 ± 14.27	7.83 ± 19.79	0.093	-30.96 (-20.14 to -41.77)	46.62 (35.80 to 57.43)
**p-value**	0.001	0.292				

n = 20 subjects per group; SD= Standard Deviation; 95% LoA= 95% Limits of Agreement; 95% CI= 95% Confidence Interval.

There was a statistically significant difference between the measurements obtained in session 1 between the two groups (unpaired t-test; p=0.001, Table 2), but no differences were found between measurements in session 2 (unpaired t-test; p=0.292, Table 2).

## DISCUSSION

DED is an endemic pathology of the tear film that is difficult to diagnose, necessitating several tests for a clear diagnosis^(1,2)^. This has resulted in a wide range of results across studies depending on the test used. Moreover, findings indicate that the most commonly used diagnostic tests for DED have poor to fair repeatability^(23)^. Hyperosmolarity stimulates the mechanisms involved in the development and progression of DED, such as elevated tear osmolarity induces apoptosis, serves as pro-inflammatory stress, and reduces the ability of mucin-like molecules to lubricate the ocular surface, which can permanently damage the ocular surface^(4,5)^. Thus, evaluation of tear film osmolarity has been proposed as a possible single marker and a useful test for tear film assessment^(4,15)^. Tears are not well characterized by a mean because a diseased tear film is an inherently chaotic unstable system characterized by rapid increases in osmolarity between blinks followed by a mixing-driven reduction to a floor, which is most likely determined by blood osmolarity^(19)^.

Tear film osmolarity was measured in two sessions one-week apart in 20 healthy and 20 DED participants, with no differences between sessions or correlation between means and differences. According to Bland and Altman, both of these characteristics are required for a clinical technique to be considered repeatable^(21)^. However, the bias range obtained during the difference analysis assessment was too high in both healthy and DED participants. Previous studies examined the variance of osmolarity in healthy participants over a single day and discovered no variation in the diurnal osmolarity pattern^(7-12,18)^. In addition, inter-day analysis over two consecutive days showed no significant differences in the measurements^(9,18)^. Those results were consistent with the current findings, demonstrating a nearly stable profile over time in the osmolarity parameter on healthy participants. Contradicting this hypothesis, several recent studies have found no variation in the diurnal osmolarity pattern in healthy participants^(7,8)^. On the other hand, some studies found no intra-diurnal variation in the osmo-larity profile in tear film-altered participants^(7)^, while others found differences between some time points^(8,9)^. In contrast to the healthy participants, previous studies found variations in the consecutive inter-day analysis.^(9)^ Those reports appear to contradict the findings of this study; DED participants exhibit variability in readings^(19)^, which is consistent with the high bias range found here despite the lack of statistical difference between sessions. All of those studies are limited by the sample size or the non-specific criteria to establish the difference between DED and healthy patients; these issues should be addressed in future studies.

Tear osmolarity provides a measurable objective numerical output, while other tests rely on subjective grading criteria^(24)^. Although osmolarity provides a rapid measurement of tear film osmometry status that can be used in clinical settings, the results here suggest that mea-surements from follow-up visits should be interpreted with caution. In the present study, there was a difference between groups in the osmolarity values obtained in the first sessions but not in the second session. In both sessions, healthy participants achieve a range of values considered “low” (301.20 ± 10.89 - 307.90 ± 11.12 mOsm/L), whereas DED participants have values higher than those considered pathological (316.13 ± 14.56 - 308.30 ± 14.27 mOsm/L). However, in one of the sessions, both groups showed a near-threshold value to be considered healthy/DED; this may imply that the DED participants enrolled here may have slightly or moderate DED, rather than a severe condition^(25)^. Moreover, tear osmolarity was found to have a low and stable profile over time in normal participants during repeated measurement, while DED participants had relatively elevated and unstable readings because the body loses control during a disease and normal homeostasis is disrupted^(15,19,26,27)^. According to Keech et al.^(19)^, it is possible to collect four consecutive measurements without significantly influencing osmolarity values in both dry eye and normal participants, with a gradual increase observed in DED participants using a short time interval. Indeed, because of the disease’s heteroscedasticity, osmolarity variability or increasing variation with increasing value is a statistical characteristic of DED participants and should be considered as a clinical indicator of the loss of tear film homeostasis^(6)^. Potvin et al.^(24)^ proposed the same hypothesis, reinforcing the idea that variability in tear osmolarity can also be a diagnostic indicator; in fact, greater inter-eye variability has been proposed as a feature that clinicians should specifically look for when diagnosing DED^(2,15,16,28)^. The current study suggests that repeated measurements over time during a clinical assessment may be more useful than an inter-week comparison.

Regarding inclusion criteria, the present study followed specific criteria to enroll two groups of subjects based on the DEWS-II diagnostic methodology report^(2)^. However, while the gender distribution was statistically similar between the two studied groups, there was a significant age difference between them. Both age and gender have been linked to DED: older patients and women are more likely to suffer from dry eyes^(29)^. According to one recent meta-analysis, the aging process has a significant impact on the ocular surface microenvironment and the existence of a tear stable physiological profile, resulting in a DED status caused by cellular senescence^(30)^. This issue could have influenced the current study’s results as a confounding factor, as it could have been the source of some variability in the mean osmolarity value between sessions in the DED group (“values above those considered pathological” in the first session and “near the threshold cut-off values” in the second).

The current findings have some limitations. First, despite the specific criteria used to enroll groups in the current study, the sample size for each group is small, with some influenced by demographics such as gender or age. Furthermore, based on the descriptive statistics obtained from the inclusions test, the DED group could be classified as “slight/moderate DED”; further research should be conducted in severe dry eye participants to obtain different results for clinical assessment. In summary, while the measurement had a high bias range, the current study found no inter-week variability in the osmolarity values of both healthy and DED participants classified using DEWS-II criteria.
